# Tuberculosis-related stigma among adults presenting for HIV testing in KwaZulu-Natal, South Africa

**DOI:** 10.1186/s12889-020-09383-0

**Published:** 2020-09-03

**Authors:** Kristina L. Bajema, Rachel W. Kubiak, Brandon L. Guthrie, Susan M. Graham, Sabina Govere, Hilary Thulare, Mahomed-Yunus Moosa, Connie Celum, Paul K. Drain

**Affiliations:** 1 Department of Medicine, University of Washington, Seattle, USA; 2 Department of Epidemiology, University of Washington, Seattle, USA; 3 Department of Global Health, University of Washington, Seattle, USA; 4 AIDS Healthcare Foundation, Durban, South Africa; 5 Department of Infectious Diseases, University of KwaZulu-Natal, Durban, South Africa

**Keywords:** Stigma, Tuberculosis, HIV, South Africa

## Abstract

**Background:**

Tuberculosis (TB)-related stigma presents a major barrier to care of persons with TB through its impact on treatment initiation and retention in care. This is particularly challenging in settings with high prevalence of both TB and HIV where fear of HIV/AIDS can amplify stigma surrounding TB. The purpose of this study was to validate a TB stigma scale for use among persons presenting for outpatient HIV screening in the Umlazi township of South Africa and evaluate factors associated with TB-related stigma in this high HIV burden setting.

**Methods:**

In this cross-sectional study, we measured TB-related stigma in adults prior to HIV testing using a 12-item scale designed to assess experienced and felt TB-related stigma.

**Results:**

Among 848 adults, mean age was 32 years, 54% were male, and the median TB stigma score was 19 of 36 (interquartile range 15–23). We identified two factors in the stigma scale which had excellent reliability (Cronbach’s alpha 0.85, 0.89). Persons with high TB stigma were more likely to be male (adjusted relative risk ratio [aRRR] 1.56, 95% confidence interval [CI] 1.11–2.28) and have accurate knowledge of TB transmission (aRRR 1.90, 95% CI 1.16–3.10) as compared to those with low stigma. Variables not significantly associated with stigma in the multivariate model included education, income, prior TB or HIV diagnoses, and depression.

**Conclusions:**

Male sex and TB knowledge were associated with higher TB stigma in an outpatient HIV clinic in a South African township. Identifying risk factors associated with stigma will be important to guide stigma reduction interventions.

## Background

In 2018, over 10 million people worldwide became ill with active tuberculosis (TB). Among persons living with HIV, TB was the leading cause of death [1]. Though there has been growing international recognition of the stigma surrounding TB, this remains a significant challenge to control of both TB and associated HIV disease [2].

Many scales have been developed to quantify HIV stigma [3], but there have been few formal tools implemented to assess TB-related stigma. Various factors associated with TB-related stigma include gender, education, socioeconomic status, and knowledge of TB transmission [4–7]. In many cases, fear of HIV/AIDS has compounded stigma surrounding TB [8, 9] and impeded HIV screening in TB patients [10, 11]. While numerous qualitative investigations have highlighted the link between TB-related stigma and delays in presentation for care [7], further studies utilizing more practical quantitative scales are needed to understand the health impacts of stigma and inform targeted interventions [12]. A TB stigma scale can be readily used in a clinical or community setting [5] as a tool to better understand the impact of stigma on care seeking behavior, treatment initiation, and retention in care. Further, given the close relationship between TB and HIV [13], an HIV clinical setting may provide a unique opportunity to measure TB-related stigma.

This study addresses the need for a quantitative measure of TB-related stigma in a setting where stigma surrounding HIV may be closely related. We used a TB stigma scale originally developed and validated in Thailand by Van Rie et al. [14] to quantify experienced and felt stigma in a large cohort of adults presenting for HIV screening in a highly endemic TB-HIV setting in South Africa. We describe sociodemographic and clinical factors associated with TB-related stigma.

## Methods

### Study design and participants

In this cross-sectional study, we consecutively enrolled adults presenting for voluntary HIV testing at the iThembalabantu Clinic in the Umlazi township of KwaZulu-Natal, South Africa between April and November 2017. Non-pregnant, antiretroviral therapy (ART) naïve adults ≥18 years of age who were able to provide written informed consent were eligible for enrollment. The study was approved by the University of Washington’s Institutional Review Board (#49563) and the University of KwaZulu-Natal’s Medical Research Ethics Committee (#BF052/13).

### Data collection and instruments

At enrollment, research assistants conducted face-to-face interviews with participants in their native English or Zulu language to obtain sociodemographic and clinical information. They also completed a 12-item questionnaire (Van Rie scale) designed to measure a patient’s experienced and felt TB-related stigma [14]. Each question was rated on a 4-point Likert scale (0-strongly disagree, 1-disagree, 2-agree, 3-strongly agree) with higher scores indicating higher levels of stigma and a maximum total score of 36. Knowledge of TB causes, symptoms, and transmission as well as participants’ perception of others in the community with TB were also ascertained by questionnaire. Depression was measured using the 9-item Patient Health Questionnaire (PHQ-9) [15], and anxiety was measured using the 7-item Generalized Anxiety Disorder (GAD-7) scale [16]. HIV counselors then performed rapid HIV testing after clinical data including assessment of TB-related stigma were collected. Those who tested HIV positive were seen by a research nurse who obtained additional medical history and blood samples for CD4 T-cell testing before initiation of ART.

### Statistical analyses

#### Validity and reliability

We excluded individuals with missing stigma responses and conducted exploratory factor analysis (EFA) using the principal factor method with oblique rotation to identify the number of factors that would emerge when the TB stigma scale was applied to our clinical setting. We used a double cross-validation approach on each randomly selected half of the study cohort to develop a model through EFA then conducted confirmatory factor analyses (CFA) on the other half of the cohort. We assessed goodness of fit by calculating the root mean square error of approximation (RMSEA: 0.08–0.10 good, < 0.05 excellent), comparative fit index (CFI: > 0.90 good, > 0.95 excellent), and Tucker and Lewis Index (TLI: > 0.90 good, > 0.95 excellent) [14]. We assessed scale reliability by calculating Cronbach’s alpha (0.70–0.80 good to excellent) [17].

#### Factors associated with TB-related stigma

We categorized TB stigma into tertiles. We compared the risk of experiencing TB stigma in the highest and middle tertiles, respectively, versus lowest tertile by clinical and sociodemographic factors using unadjusted multinomial logistic regression models. Baseline factors found to be significantly associated with TB stigma in univariate models (*P* ≤ 0.10) were included in the multivariate model. Results are presented as relative risk ratios (RRR) and 95% confidence intervals (CI). We used multiple imputations by chained equations to perform 50 imputations of prior positive HIV test, the only variable for which ≥5% of the data were missing.

#### Sensitivity analysis

We explored the robustness of our findings to different classification approaches for ‘low,’ ‘moderate,’ and ‘high’ TB stigma. We first considered an alternative grouping based on scoring 0–12, 13–24, and 25–36 respectively. The original questionnaire scores each item from 0 to 3, assuming an even difference between responses ‘strongly disagree,’ ‘disagree,’ ‘agree,’ and ‘strongly agree.’ We further explored the impact of uneven score differences between item responses by using a scoring of 0, 1, 3, 4 (greater difference between disagree and agree) and 0, 2, 3, 5 (greater difference between strongly disagree/agree and disagree/agree) categorized into tertiles. Analysis was performed using Stata, version 14 (StataCorp, College Station, TX).

## Results

We screened 862 adults. After excluding 14 participants who did not complete all TB-related stigma questionnaire items, we included 848 participants for analysis. Of these, 445 (52%) tested HIV positive. Mean age was 32 years, 454 (54%) were men, 158 (19%) had a partner with HIV, 221 (29%) had a prior positive HIV test, and 57 (7%) had a prior positive TB test (Table 1). Among persons who tested HIV positive at enrollment, median CD4 was 339 cells/mm^3^ (interquartile range [IQR] 190–524 cells/mm^3^).

**Table 1:** Sociodemographic and clinical characteristics of study participants

Characteristic	Total *N* = 848
*Demographics*	
Age, mean^a^	32 (10)
Male^b^	454 (54)
Ethnicity	
Zulu	805 (95)
Xhosa	27 (3)
Other	12 (1)
Education	
Less than high school	304 (36)
High school or higher degree	543 (64)
Marital status	
Married	35 (4)
Never married	806 (95)
Widowed/divorced	7 (1)
Have children	582 (70)
Ever used tobacco	192 (23)
Ever consumed alcohol	369 (44)
Partner’s HIV status	
Negative	275 (32)
Positive	158 (19)
Unknown	414 (49)
Currently employed	
No	497 (59)
Yes, working ≤20 h per week	298 (35)
Yes, working > 20 h per week	53 (6)
< 2000 ZAR/month income^c^	504 (60)
< 5 km distance to nearest clinic	585 (69)
Internet use^d^	654 (77)
Social media use^e^	637 (75)
*Clinical*	
Depression (PHQ-9 ≥ 5)	71 (8)
Anxiety (GAD-7 ≥ 5)	57 (7)
HIV	
Prior positive HIV test	221 (29)
HIV positive at enrollment	445 (52)
Median CD4 among positive, cells/mm^3^ (IQR)	339 (190–524)
TB	
Prior positive TB test	57 (7)
TB symptoms^f^	
Cough > 2 weeks	31 (7)
Cough < 2 weeks	56 (13)
No cough	356 (80)
Fever	68 (15)
Weight loss	129 (29)
Night sweats	87 (20)

^a^Mean values followed by standard deviation

^b^Count values followed by percentage

^c^1 ZAR = 0.08 US dollars in 2017

^d^Indicated internet use at home, work, school, family or friend’s house, or community center

^e^Included use of Facebook, Twitter, or WhatsApp

^f^Symptoms ascertained in HIV positive individuals only

*Abbreviations*: *GAD-7* Generalized Anxiety Disorder-7, *km* kilometers, *PHQ-9* Patient Health Questionnaire-9, *TB* tuberculosis, *ZAR* South African rand

Accurate knowledge of the route of TB transmission was good (84%) while awareness of a pathogen being the cause was lower (40%, Table 2). Community perspectives toward TB were generally suggestive of high levels of stigma. While most (809 persons, 96%) did not feel that a family member’s diagnosis of TB should remain a secret, 214 (25%) indicated feeling sorry for persons with TB disease and that they tended to avoid them. Moreover, 89 (11%) indicated they would not be willing to work with someone previously treated for TB and only 74 (9%) indicated that their community was supportive of persons with TB.

**Table 2:** Knowledge and community perspectives toward tuberculosis among study participants

Characteristic	Total *N* = 848
*TB knowledge, N (%)*	
Awareness of cause^a^	335 (40)
Awareness of transmission^b^	713 (84)
*Community perspectives toward TB, N (%)*	
If a member of your family got TB, would you want it to remain a secret or not?	
Yes, remain a secret or confidential	22 (3)
No	809 (96)
Don’t know	11 (1)
Would you be willing to work with someone who has been previously treated for TB?	
Yes	756 (89)
No	89 (11)
Which statement is closest to your feeling about people with TB disease	
I feel sorry for them but I would like to help them.	610 (72)
I feel sorry for them but I tend to stay away from these people.	214 (25)
It is their problem and I cannot get TB.	7 (< 1)
I fear them because they may infect me.	16 (2)
I have no particular feeling.	1 (< 1)
In your community how is a person with TB usually regarded/treated?	
Most people reject him or her.	391 (47)
Most people are friendly but they generally try to avoid him or her.	370 (44)
The community mostly supports him or her.	74 (9)

^a^Correctly identified the cause of TB as microbes, germs, or bacteria

^b^Correctly identified that TB is transmitted through the air

*Abbreviation*: *TB* tuberculosis

### Validity and reliability of the TB stigma scale

There was considerable variability in the frequency of responses to the 12 stigma questions: strongly disagree (0.2–11.5%), disagree (19.0–72.2%), agree (22.2–48.9%), strongly agree (2.8–43.8%). In sampling the first half of the cohort, we identified a single factor with eigenvalue > 1.0 which accounted for 48% of the total variance in the scale and had good factor loadings of 0.56–0.78 (Table 3). Goodness-of-fit in cross-validation was poor across all indices: RMSEA 0.18, CFI 0.74, TLI 0.69. Cronbach’s alpha was excellent (0.91). In sampling the second half of the cohort, we identified two factors with eigenvalues > 1.0 which accounted for 56% of the total variance in the scale. The first factor contained six items with rotated factor loadings of 0.53 to 0.94 while the second factor contained six items with rotated factor loadings of 0.49 to 0.93. Goodness-of-fit in cross-validation was improved across all indices: RMSEA 0.11, CFI 0.90, TLI 0.87. After eliminating two questions with factor loadings < 0.50 [18], fit was further improved for both CFI (0.92) and TLI (0.90). Cronbach’s alpha was excellent for each of the two subscales (0.85, 0.89).

**Table 3:** Tuberculosis stigma scale and factor loadings^a^

	Factor 1^b^	Factor 1^c^	Factor 2
1. People who have TB feel hurt of how others react to knowing they have TB.	0.66		0.90
2. People who have TB lose friends when they share with them they have TB.	0.68		0.80
3. People who have TB feel alone.	0.70		0.70
4. People who have TB keep their distance from others to avoid spreading TB germs.	0.65		0.49
5. People who have TB are afraid to tell those outside their family that they have TB.	0.74		0.49
6. People who have TB are afraid of going to TB clinics because other people may see them there.	0.68	0.53	
7. People who have TB are afraid to tell others that they have TB because others may think that they also have AIDS.	0.73	0.57	
8. People who have TB feel guilty because their family has the burden of caring for them.	0.67	0.68	
9. People who have TB will choose carefully who they tell about having TB.	0.56		0.52
10. People who have TB feel guilty for getting TB because of their smoking, drinking, or other careless behavior.	0.63	0.82	
11. People who have TB are worried about having AIDS.	0.78	0.88	
12. People who have TB are afraid to tell their family that they have TB.	0.77	0.94	

^a^Each question is rated on a 4-point Likert scale: 0 - strongly disagree, 1 - disagree, 2 - agree, 3 - strongly agree. Maximum score 36

^b^A single factor structure is observed for exploratory factor analysis on the first half of the cohort

^c^A two factor structure is observed on the second half of the cohort

*Abbreviation: TB* tuberculosis

### TB-related stigma among participants

The median TB stigma score at baseline was 19 (IQR 15–23). Tertile one ranged from 0 to 16, tertile two from 17 to 21, and tertile three from 22 to 36. In univariate models, factors associated with higher TB stigma included male sex, being unmarried, having employment and higher income, and accurate knowledge of TB causes and transmission. Having an HIV-positive partner was associated with lower TB stigma. In the multivariate model, male sex was associated with a higher risk of being in the 3rd versus 1st stigma tertile (aRRR 1.59, 95% CI 1.11–2.28, *P* 0.01) while knowledge of TB transmission was associated with a higher risk (aRRR 1.90, 95% CI 1.16–3.10, *P* 0.01, Table 4).

**Table 4:** Relationship of clinical and sociodemographic factors with the risk of perceiving high or moderate versus low tuberculosis stigma^a^

	Unadjusted Models				Adjusted Model^b^			
	2nd Tertile		3rd Tertile		2nd Tertile		3rd Tertile	
	RRR (95% CI)	*P* Value	RRR (95% CI)	*P* Value	aRRR (95% CI)	*P* Value	aRRR (95% CI)	*P* Value
*Demographics*								
Age, years	0.99 (0.97–1.01)	0.24	0.99 (0.98–1.01)	0.43	0.99 (0.97–1.01)	0.52	0.99 (0.97–1.01)	0.42
Male	1.25 (0.90–1.74)	0.19	1.62 (1.17–2.25)	< 0.01	1.39 (0.96–2.02)	0.08	1.59 (1.11–2.28)	0.01
High school education or higher	0.97 (0.69–1.37)	0.87	1.01 (0.72–1.42)	0.94	–		–	
Unmarried	2.17 (0.89–5.28)	0.09	1.64 (0.74–3.62)	0.22	2.07 (0.77–5.55)	0.15	1.53 (0.66–3.56)	0.32
Has children	0.75 (0.53–1.08)	0.13	0.86 (0.60–1.23)	0.42	–		–	
Ever used tobacco	1.06 (0.71–1.57)	0.78	1.06 (0.72–1.56)	0.78	–		–	
Ever used alcohol	1.14 (0.82–1.60)	0.44	1.23 (0.88–1.70)	0.22	–		–	
Partner’s HIV status								
Negative	Ref		Ref		Ref		Ref	
Positive	0.63 (0.38–1.04)	0.07	0.66 (0.42–1.04)	0.07	0.64 (0.38–1.08)	0.09	0.69 (0.43–1.12)	0.13
Unknown	1.10 (0.75–1.61)	0.62	0.79 (0.49–1.02)	0.07	1.20 (0.80–1.80)	0.39	0.82 (0.55–1.21)	0.31
Employed	1.05 (0.75–1.47)	0.79	1.40 (1.01–1.95)	0.04	0.89 (0.42–1.90)	0.76	0.81 (0.39–1.68)	0.58
Monthly income ≥2000 ZAR	1.13 (0.80–1.59)	0.48	1.54 (1.11–2.15)	0.01	1.16 (0.54–2.51)	0.70	1.61 (0.77–3.78)	0.21
Distance to nearest clinic ≥5 km	0.61 (0.42–0.89)	< 0.01	1.02 (0.73–1.44)	0.89	0.68 (0.46–1.02)	0.07	1.11 (0.77–1.61)	0.57
Internet use	1.45 (0.98–2.16)	0.07	1.32 (0.90–1.93)	0.16	1.38 (0.53–3.60)	0.51	0.53 (0.18–1.51)	0.23
Social media use	1.38 (0.94–2.03)	0.10	1.42 (0.98–2.06)	0.07	1.11 (0.44–2.80)	0.82	2.58 (0.92–7.21)	0.07
*Clinical*								
Depression, PHQ-9 ≥ 5	1.45 (0.79–2.67)	0.23	1.44 (0.79–2.62)	0.24	–		–	
Anxiety, GAD-7 ≥ 5	0.91 (0.48–1.72)	0.77	0.68 (0.35–1.33)	0.26	–		–	
Prior positive HIV test^c^	1.24 (0.86–1.80)	0.25	0.81 (0.55–1.18)	0.27	–		–	
Prior positive TB test	0.85 (0.42–1.71)	0.65	1.31 (0.71–2.45)	0.39	–		–	
HIV positive at enrollment	1.19 (0.86–1.67)	0.30	0.80 (0.58–1.10)	0.17	–		–	
*TB Knowledge*								
Awareness of cause	1.39 (0.98–1.96)	0.07	1.11 (0.79–1.56)	0.54	1.33 (0.93–1.91)	0.12	1.08 (0.76–1.54)	0.68
Awareness of transmission	1.35 (0.85–2.15)	0.20	1.62 (1.01–2.60)	0.04	1.38 (0.84–2.25)	0.20	1.90 (1.16–3.10)	0.01

^a^Stigma is categorized into tertiles: low (0–16, *N* = 313), moderate (17–21, *N* = 256), high (22–36, *N* = 279)

^b^Adjusted for age, sex, marital status, partner’s HIV status, employment, income, distance to nearest clinic, internet use, social media use, knowledge of TB cause and transmission. Total *N* = 793

^c^Imputed results are presented

*Abbreviations*: *aRRR* adjusted relative risk ratio, *CI* confidence interval, *GAD-7* Generalized Anxiety Disorder-7; *km* – kilometers, *RRR* relative risk ratio, *PHQ-9* Patient Health Questionnaire-9, *ZAR* South African rand

In sensitivity analyses using different classification approaches for stigma groups, these patterns were similar with a few exceptions. When classifying by a ‘low’ (0–12) (*N* = 93, 11%), ‘moderate’ (13–24) (*N* = 585, 69%) and ‘high’ (25–36) (*N* = 170, 20%) method, sex was no longer significantly associated with stigma (aRRR ‘moderate’ 1.15, 95% CI 0.67–1.99, *P* 0.60; aRRR ‘high’ 1.52, 95% 0.81–2.84, *P* 0.19), while being unmarried reached statistical significance (aRRR ‘moderate’ 7.56, 95% CI 2.99–19.10, *P* < 0.01; aRRR ‘high’ 5.23, 95% CI 1.71–15.96, *P* < 0.01). Accurate knowledge related to TB transmission was even more strongly associated with ‘high’ stigma (aRRR 2.39, 95% CI 1.18–4.84, *P* 0.02) and reached statistical significance for ‘moderate’ stigma (aRRR 2.17, 95% CI 1.22–3.85, *P* < 0.01). When classifying by uneven scoring of 0, 1, 3, 4, the magnitude of association between male sex and stigma was similar to the main analysis and significant in both groups (aRRR ‘moderate’ 1.52, 95% CI 1.05–2.19, *P* 0.03; aRRR ‘high’ 1.52, 95% CI 1.06–2.19, *P* 0.02). Results for knowledge around TB transmission were also similar. Living farther from clinic was associated with significant lower risk of ‘moderate’ stigma (aRRR 0.65, 95% CI 0.43–0.98, *P* 0.04). When classifying by uneven scoring of 0, 2, 3, 5, knowledge of TB transmission was no longer significantly associated with increased stigma while results for sex were similar.

## Discussion

We applied a validated TB stigma scale in our cohort of adults presenting for HIV screening in a high TB-HIV burden setting in South Africa. In this clinical setting, we identified one to two factors in the scale which had good reliability. We found that men and persons knowledgeable about TB transmission reported higher levels of stigma. Meanwhile, education, income, prior TB or HIV diagnoses, and depression were not significantly associated with stigma our analysis.

Few studies have evaluated quantitative TB stigma scales in sub-Saharan Africa, and there is currently no validated scale in routine use among South African patients presenting for TB or HIV care [19–23]. In this study, we report application of the Van Rie scale in the largest cohort to date [14, 24–26]. We investigated a number of sociodemographic and clinical factors potentially associated with experienced and felt stigma surrounding TB.

Globally, there is cultural variation in how men and women experience TB-related stigma. Factors related to sex differences in stigma include differences in financial dependence and social isolation by gender and the often greater impact of TB on marital prospects for women [4, 7]. Research with focus group discussions and in-depth interviews can inform how stigma is felt and perceived differently by men and women and why men presenting for HIV screening in urban South Africa experienced higher TB-related stigma.

TB knowledge has also been linked to stigma. Among TB patients in China, TB knowledge encompassing route of transmission, symptoms, and curability was associated with lower stigma [27]. When knowledge of TB transmission and knowledge of TB curability were examined separately across multiple general population surveys, knowledge of TB transmission was positively associated with stigma while knowledge of curability was negatively associated [6]. TB knowledge is complex, and differences in the definition of knowledge as well as the type of instrument used to measure stigma make it difficult to compare findings across studies.

Stigma surrounding TB and HIV is often closely linked, and HIV coinfection has been associated with greater perceived TB-related stigma [8, 9]. In our cohort, we did not find that a prior positive HIV test or HIV status at enrollment were associated with stigma. This may be due to selection of participants willing to present for HIV screening who may experience less HIV-related stigma than individuals who do not seek HIV screening.

We applied a stigma scale designed to measure patients’ experience of TB-related stigma in a setting where participants were primarily presenting for HIV rather than TB screening; it is therefore important to interpret the psychometric properties of the scale in this context. While we assumed a priori that a single factor, “patient perspectives toward TB,” would be identified as was found when the scale was originally validated, a one to two factor model emerged in our analysis. Conceptually, three types of stigma have been identified in the HIV literature: enacted, anticipated, and internalized stigma [3]. Similar subdomains of TB-related stigma may be observed here with a dichotomy between enacted and anticipated/internalized stigma. Construct validity was limited in the single factor model but improved in the two factor model. This underscores the challenge of adapting scales to different cultural settings and clinical contexts. The questionnaire ascertaining community perspectives toward TB revealed high levels of stigma and may be a more appropriate instrument in an HIV-focused clinic setting. Future work in similar settings may therefore benefit from a community-focused approach to measuring stigma. Finally, a more complete assessment of stigma in this setting will require focus group discussions, in-depth interviews, and qualitative analysis to fully validate a stigma scale and better understand the meaning of TB stigma scores.

Previous experience of TB disease can shape an individual’s perception of stigma, and we were not able to fully measure this history in our study. While we observed that 7% of participants had a prior positive TB test, we did not asses how many were previously screened. Given the longstanding recommendation to screen all persons with HIV for TB in South Africa [28] and report of 29% of participants having a prior positive HIV test, we infer that prior TB screening experience was common. Further, we did not assess how many participants had a family member with TB.

As there are no established criteria on ‘low,’ ‘moderate,’ or ‘high’ stigma, we developed our own classification. While there was some variation in associated factors in sensitivity analyses, gender and TB knowledge were generally robust to different categorization approaches. Our finding that knowledge of TB transmission was associated with higher levels of stigma is important to consider when designing TB-related stigma reduction interventions. Previously studied interventions have often focused on knowledge-shaping efforts, and results have been variable [12]. In one study, volunteers were trained over two days to provide TB education in their community which led to improved knowledge including mode of transmission but worsened stigmatizing attitudes [29]. Knowledge can either enhance stigma due to fear of acquiring TB from others or reduce stigma by supporting a more positive outlook due to awareness of treatment and cure [6, 30]. It is therefore important to ensure comprehensive messaging and ongoing training to address any misconceptions around TB disease.

## Conclusions

We validated a TB stigma scale designed to quantify an individual’s experienced and felt TB-related stigma in a highly TB-HIV endemic outpatient clinical setting in a South African township. We examined a number of clinical and sociodemographic characteristics and found male sex and knowledge of TB transmission were consistently associated with greater TB-related stigma. These findings can be informative to clinicians and policy makers seeking to improve TB and HIV care through implementation of knowledge-shaping interventions [12] tailored to gender differences in experienced and felt TB-related stigma.

## Data Availability

The dataset used in this study is available from the corresponding author on reasonable request.

## Abbreviations

aRRR: Adjusted relative risk ratio
ART: Antiretroviral therapy
CFA: Confirmatory factor analysis
CFI: Comparative fit index
CI: Confidence interval
EFA: Exploratory factor analysis
GAD-7: Generalized Anxiety Disorder-7
IQR: Interquartile range
PHQ-9: Patient Health Questionnaire-9
RMSEA: Root mean square error of approximation
RRR: Relative risk ratio
TB: Tuberculosis
TLI: Tucker and Lewis Index
